# The Identification and Characteristics of Immune-Related MicroRNAs in Haemocytes of Oyster *Crassostrea gigas*

**DOI:** 10.1371/journal.pone.0088397

**Published:** 2014-02-06

**Authors:** Zhi Zhou, Lingling Wang, Linsheng Song, Rui Liu, Huan Zhang, Mengmeng Huang, Hao Chen

**Affiliations:** 1 Key Laboratory of Experimental Marine Biology, Institute of Oceanology, Chinese Academy of Sciences, Qingdao, China; 2 University of Chinese Academy of Sciences, Beijing, China; Uppsala University, Sweden

## Abstract

**Background:**

MicroRNAs (miRNAs) represent a class of small ncRNAs that repress gene expression on the post-transcriptional level by the degradation or translation inhibition of target mRNA.

**Methodology:**

Three small RNA libraries from oyster haemocytes were sequenced on the Illumina platform to investigate the latent immunomodulation of miRNAs after bacteria challenge and heat stress. Totally, 10,498,663, 8,588,606 and 9,679,663 high-quality reads were obtained in the control, bacteria and bacteria+heat library, respectively, from which 199 oyster miRNAs including 71 known and 128 novel ones were identified. Among these miRNAs, 6 known and 23 novel ones were predicted to possess more than one precursor-coding region, and cgi-miR-10a, cgi-miR-184b, cgi-miR-100, cgi-miR-1984 and cgi-miR-67a were observed to be the most abundant miRNAs in the control library. The expression levels of 22 miRNAs in the bacteria library were significantly higher than those in the control library, while there were another 33 miRNAs whose expression levels were significantly lower than that in the control library. Meanwhile, the expression levels of 65 miRNAs in the bacteria+heat library changed significantly compared to those in the bacteria library. The target genes of these differentially expressed miRNAs were annotated, and they fell in immune and stress-related GO terms including antioxidant, cell killing, death, immune system process, and response to stimulus. Furthermore, there were 42 differentially expressed miRNAs detected in both control/bacteria and bacteria/bacteria+heat comparisons, among which 9 miRNAs displayed the identical pattern in the two comparisons, and the expression alterations of 8 miRNAs were confirmed using quantitative RT-PCR.

**Conclusions:**

These results indicated collectively that immune challenge could induce the expression of immune-related miRNAs, which might modulate the immune response such as redox reaction, phagocytosis and apoptosis, and the expression of some immune-related miRNAs could be also regulated by heat stress to improve the environmental adaption of oyster.

## Introduction

MicroRNAs (miRNAs) are endogenously encoded single-stranded non-coding RNAs of about 22 nt in length [1]. They are initially transcribed by RNA polymerase II in the nucleus as primary miRNAs, which are cleaved by the nuclear RNase III type enzyme Drosha to produce a short hairpin precursor miRNA. After transferring into the cytoplasm, the precursor miRNA is further cleaved by Dicer into the functional double-stranded RNA, which is incorporated into the RNA-induced silencing complex (RISC) and forms the mature miRNA [2], [3]. So far, a large number of miRNAs have been identified in various metazoans, many of which are evolutionarily conserved, and have evolved to be potent regulators of gene expression on the post-transcriptional level [4].

Mature miRNAs have the ability to regulate the stability and/or translational efficiency of their mRNA targets in metazoa through the imperfect base-pairing between target transcript and the 5′ seed region of the miRNA [5]. It has been reported that more than 60% of mammalian protein-coding genes are computationally predicted as targets of miRNA [6]. In addition, it has been considered that one gene can contain multiple miRNA binding sites, and one miRNA can regulate hundreds of target mRNAs, resulting in a complex gene-regulatory network to implement the spatio-temporal coordination of gene expression under specific development stage or physiological status [1], [5], [7]. The miRNA-coordinated gene expression contributes to the maintenance of homeostasis and the improvement of host adaption [8].

As a regulator of gene expression on the post-transcriptional level, miRNAs play an important role in the modulation of many biological processes to confer robustness on these biological processes, and further maintain the tissue identity in a variety of metazoans [9]. It has been evidenced that miRNAs are able to modulate host immune and stress responses [8], [10]–[13]. The expression of immune-related miRNAs in immunocytes can be regulated by the immune response against the invasive pathogens [14], and then these miRNAs can modulate properly the expression of pattern recognition receptors, signal pathway molecules or immune transcription factor to regulate the host-pathogen interaction and the elimination of invasive pathogens [15]–[18]. For example, mammalian NF-κB signal pathway is modulated dynamically by a set of miRNAs (including let-7, miR-9, miR-21, miR-218, and so on) during the whole process of immune response [16], [19]. In addition, stress response can also alter the biogenesis of miRNAs [20], and then the miRNAs function as the buffer to attenuate the harmful effect of stresses on some physiological activities [11], [21]. Mollusca are a large and diverse phylum in invertebrates, and there are several reports about the identification of miRNAs in some species. To our knowledge, there are 5 miRNAs identified from *Haliotis rufescens* and 60 miRNAs from *Lottia gigantean* [22]. However, there is still no any report about the physiological regulation function of mollusc miRNAs, specially their immunomodulation.

Because of the economic and ecological importance of Pacific oyster *Crassostrea gigas,* it has become a suitable model organism for studying immune and stress response in marine bivalves [23], [24]. Furthermore, the recent released genome sequence provides the relatively complete genetic information for the exploration of physiological function of oyster miRNAs [25]. Investigations of miRNAs in oyster *C. gigas* will pave a new way to further understand the modulation mechanism of immune-related genes in molluscs during the immune and stress response. The purposes of this study were to (1) identify the known and novel miRNAs from oyster *C. gigas*, (2) survey the expression alteration of all identified miRNAs in haemocytes of oyster after bacteria challenge and heat stress, (3) predict the target genes of miRNAs, and analyze GO information of the target genes of differentially expressed miRNAs to understand the potential immunomodulation of miRNAs in oyster.

## Result

### Overview of Small RNA Library Sequencing

**Figure 1:**
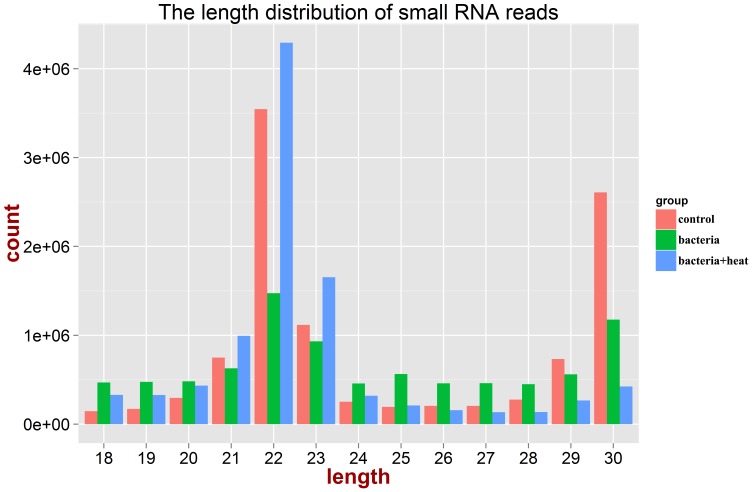
Length distribution of high-quality reads in the three libraries. Red: control library; Green: bacteria library; Blue: bacteria+heat library.

The small RNA libraries from the haemocyte samples in the control, bacteria and bacteria+heat groups were sequenced by Illumina deep sequencing technology to survey the miRNA transcriptome in oyster. Totally 11,760,772 raw reads were obtained from the control library, 11,361,851 from the bacteria library, and 12,165,062 from the bacteria+heat library. After the discarding of low-quality sequence, adaptor sequence and sequences shorter than 18 nt and longer than 30 nt, 10,498,663, 8,588,606 and 9,679,663 high-quality reads were remained for the statistics analysis of sequence length (Table 1), and 51.6%, 35.3% and 71.7% reads were of 21∼23 nt in the control, bacteria and bacteria+heat library, respectively (Fig. 1).

**Table 1:** Statistics for the distribution of reads filtered in order.

	Reads in the control group	Reads in the bacteira group	Reads in the bacteria+heat group
Total	11760772	11361851	12165062
Low quality filter	317263 (2.7%)	427064 (3.8%)	339547 (2.8%)
Adaptors filter	68014 (0.6%)	30211 (0.3%)	51019 (0.5%)
Length filter	876832 (7.5%)	2315970 (20.4%)	2094833 (17.2%)
Remaining reads	10498663	8588606	9679663

After the merging of reads from three libraries and the removal of redundancy, 229,302 clean sequences with more than 3 reads were retained for alignment analysis. After comparing the clean RNA sequences with the Rfam databases, Repbase databases and oyster mRNAs, a total of 80,005 sequences derived from other non-coding RNA, repeat sequence or mRNA degradation product were removed (Table 2). The remaining 149,297 sequences were retained for further miRNA identification analysis.

**Table 2:** Statistics for the filtered clean reads.

	Remaining Reads	
Total	28766932	
Remove redundancy	2345828	
Less than 3 reads	229302	
Hit Rfam and Repbase	194881	
Hit mRNA	149297	
Mapped miRNA	2292	

### The Discovery Of known and Novel miRNAs in Oyster

**Figure 2:**
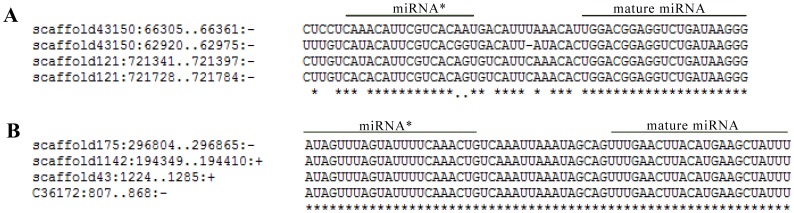
Precursor sequence alignment of miRNAs having four potential coding regions. (A) miR-184a; (B) scaffold175_3234.

To identify known miRNAs in oyster, 149,297 filtered unique sequences were aligned against mature miRNAs in miRBase (version 19), and 2292 sequences homologous to registered mature miRNAs (with not more than one mismatch between sequences) were obtained. These homologous sequences were mapped to oyster genome sequence and further parsed through the miRDeep2 software for the prediction of precursor sequence and secondary structure. A total of 71 known miRNAs were identified in oyster (Table S1) with copy numbers ranging from 0 (cgi-miR-242) to 1,126,609 (cgi-miR-10a) in the control library (Table S3). The sequences that did not match registered mature miRNAs were also aligned with the oyster genome sequence to discover potential novel miRNAs, and total of 128 novel miRNAs were identified by miRDeep2 software (Table S2) with copy numbers ranging from 0 (scaffold888_4140, scaffold347_3876, scaffold1827_3866, scaffold43878_3031, scaffold35684_4427, scaffold531_824, scaffold42948_2113, scaffold535_3052, scaffold1174_3059, scaffold1769_5047) to 38,193 (scaffold42648_5080) in the control library (Table S3). In total, 199 oyster miRNAs were identified including 71 known and 128 novel ones, and the nucleotide sequences and genome coding regions of their precursors were shown in Table S1 and S2. Among these 199 oyster miRNAs, 6 known and 23 novel ones were observed to have more than one precursor-coding region. Furthermore, there were four potential precursors in the oyster genome for cgi-miR-184a, cgi-miR-184d and scaffold175_3234 (Fig. 2).

### Differentially Expressed miRNAs during Bacteria Challenge and Heat Stress

**Figure 3:**
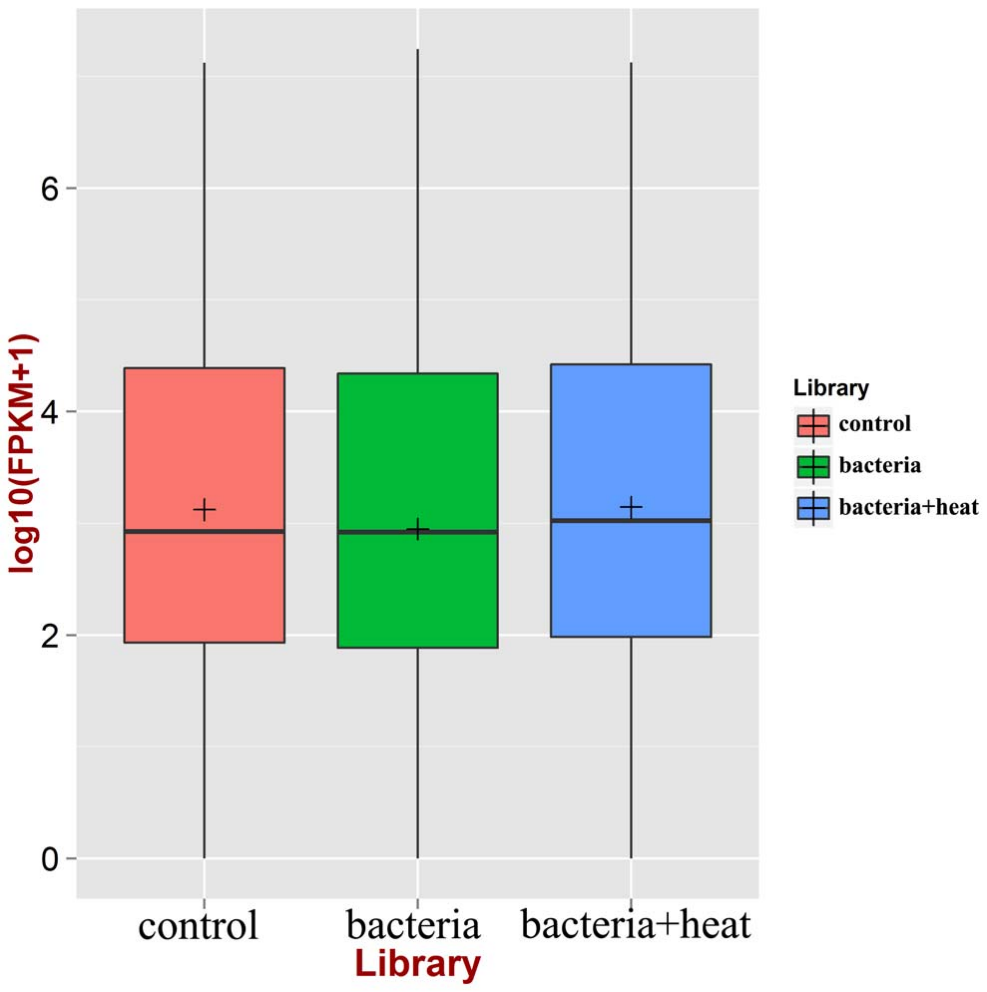
The overall expression level of all identified miRNAs in three libraries. Red: control library; Green: bacteria library; Blue: bacteria+heat library. The overall expression level of oyster miRNAs in the bacteria+heat and bacteria library was highest and lowest, respectively.

In the control library, 5 miRNAs including cgi-miR-10a, cgi-miR-184b, cgi-miR-100, cgi-miR-1984, cgi-miR-67a were identified with higher copy numbers, and their copy numbers were 1,126,609, 957,131, 308,405, 234,455 and 179,096, respectively. The copy numbers of oyster miRNAs were also counted in the control, bacteria and bacteria+heat library, respectively, and further converted to their expression levels in the form of FPKM (Table S3). The expression level of all oyster miRNAs in the three libraries was analyzed and shown in Fig. 3. The overall expression level of oyster miRNAs in the bacteria library was lower than that in the control group, while the level in the bacteria+heat library was higher than that in the control and bacteria libraries.

**Figure 4:**
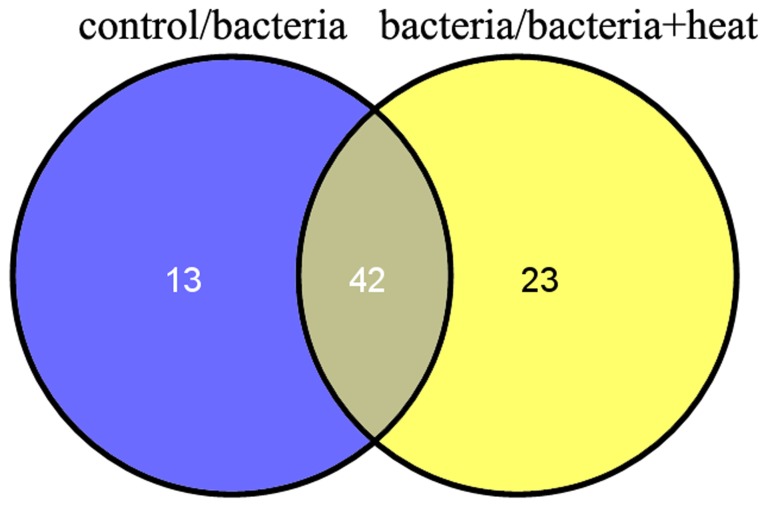
Grouping of differentially expressed miRNAs between two groups among the control, bacteria and bacteria+heat library.

**Figure 5:**
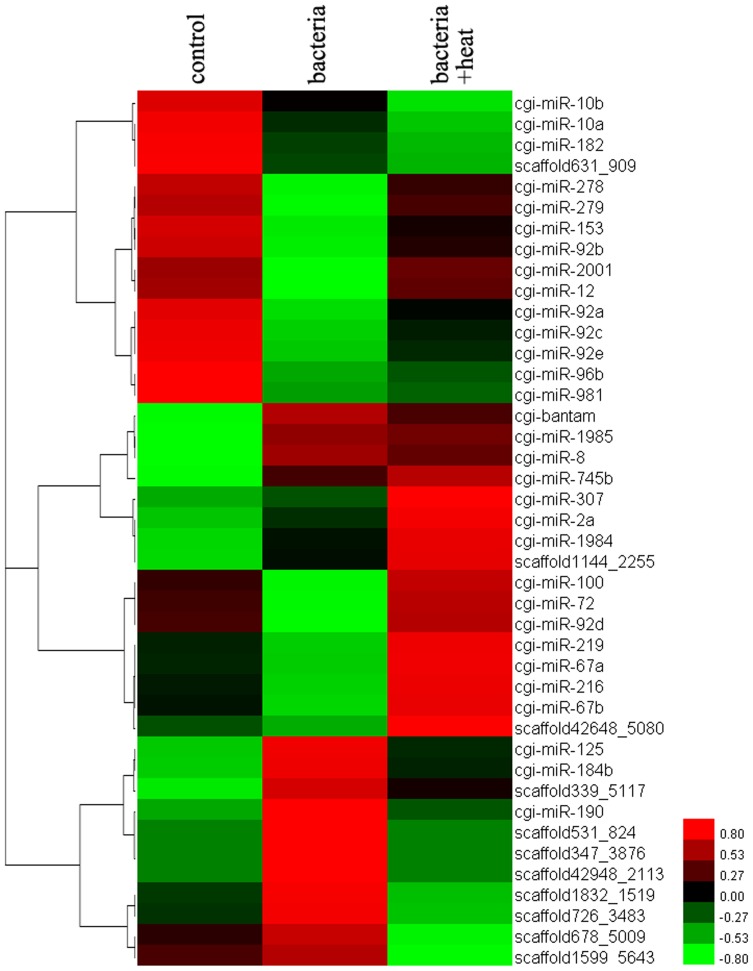
Summary cluster map of miRNAs expression. The miRNAs all changed significantly between two groups among the control, bacteria and bacteria+heat library. There was the identical alteration trend in the expression level of 9 miRNAs (cgi-miR-2a, cgi-miR-307, cgi-miR-745b, cgi-miR-1984, scaffold1144_2255, cgi-miR-10a, cgi-miR-10b, cgi-miR-182 and scaffold631_909) between the control/bacteria and bacteria/bacteria+heat comparisons. In addition, the alteration trend of other 33 miRNAs expression was contrary between the two comparisons.

Based on the statistical analysis of copy numbers, the expression of 55 miRNAs in the bacteria library changed significantly compared to that in the control group, among which 22 and 33 ones increased and decreased significantly, respectively. Meanwhile, the expression levels of 65 miRNAs in the bacteria+heat library also changed significantly (37 miRNAs increased and 28 miRNAs decreased) compared to that in the bacteria library (Table S4). Furthermore, the expression of 42 miRNAs altered significantly in both control/bacteria and bacteria/bacteria+heat comparisons (Fig. 4). Among the 42 miRNAs, the expression level of 5 miRNAs (cgi-miR-2a, cgi-miR-307, cgi-miR-745b, cgi-miR-1984 and scaffold1144_225) increased significantly in both two comparisons, while the expression level of 4 miRNAs (cgi-miR-10a, cgi-miR-10b, cgi-miR-182 and scaffold631_909) decreased significantly (Fig. 5).

### The Confirmation of Differentially Expressed miRNAs

**Figure 6:**
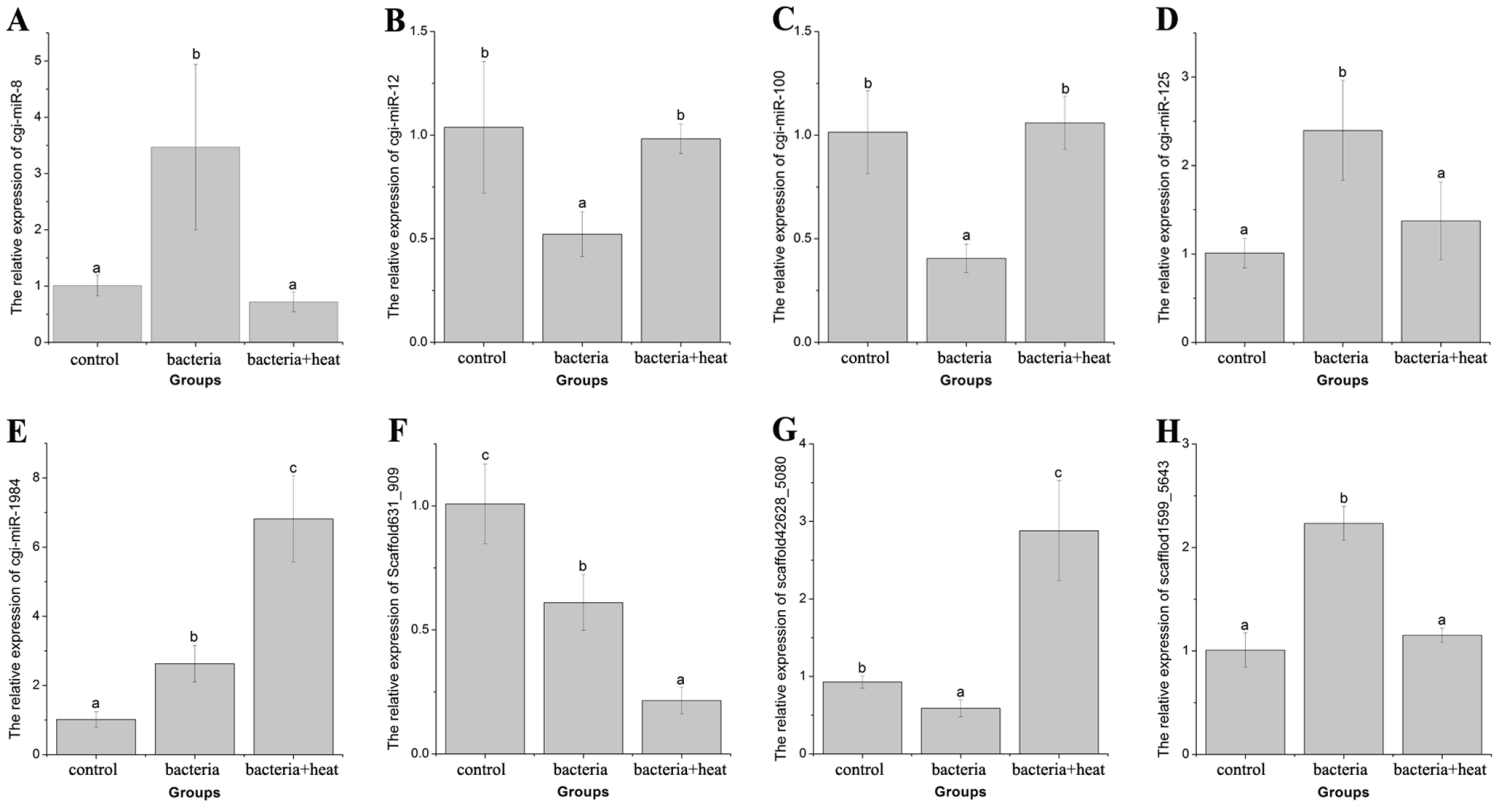
The expression of eight miRNAs detected by SYBR Green real-time PCR in oyster haemocytes after bacteria challenge and heat stress, including cgi-miR-8 (A), cgi-miR-12 (B), cgi-miR-100 (C), cgi-miR-125 (D), cgi-miR-1984 (E), scaffold631_909 (F), scaffold42648_5080 (G) and scaffold1599_5643 (H). 5S gene was used as an internal control to calibrate the cDNA template for all the samples. Each values were shown as mean ± SD (N = 5), and bars with different letters were significantly different (*P*<0.05).

The expression of eight differentially expressed miRNAs in both control/bacteria and bacteria/bacteria+heat comparisons, including cgi-miR-8, cgi-miR-12, cgi-miR-100, cgi-miR-125, cgi-miR-1984, scaffold631_909, scaffold42648_5080 and scaffold1599_5643 (Fig. 6 A–H), were confirmed by quantitative real-time PCR. The expression alterations of the eight miRNAs were consistent between the small RNA sequencing and quantitative real-time PCR. The expression level of cgi-miR-8, cgi-miR-125, cgi-miR-1984 and scaffold1599_5643 increased significantly (*P*<0.05), whereas the expression level of other four miRNAs decreased significantly (*P*<0.05) in oyster haemocytes of the bacteria group, relative to that in the control group. Furthermore, cgi-miR-1984 expression level also increased significantly (*P*<0.05) in oyster haemocytes of the bacteria+heat group compared to that in the bacteria group (Fig. 6E), and scaffold631_909 expression level also decreased significantly (*P*<0.05) (Fig. 6F). However, the expression level of other six miRNAs was reverted in oyster haemocytes of the bacteria+heat group in comparison to that in the bacteria group.

### GO Analysis of Target Genes of Differently Expressed miRNAs

**Figure 7:**
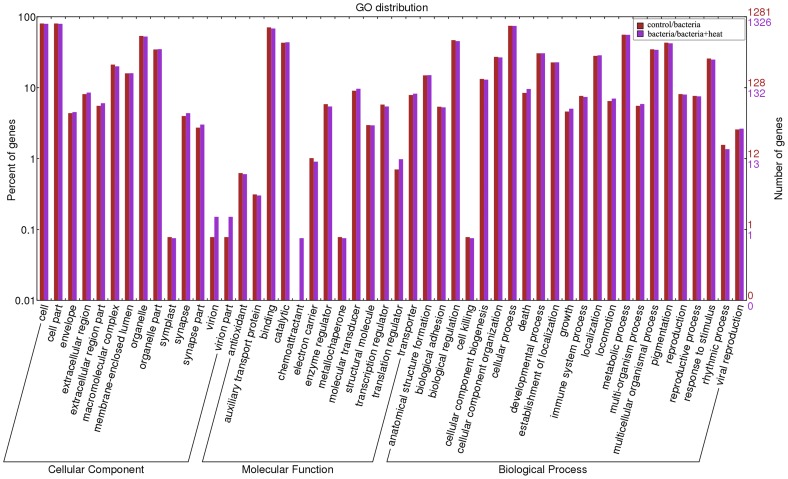
GO distribution of target genes of differentially expressed miRNAs. Red: the target genes of differentially expressed miRNAs between the control and bacteria group; Purple: the target genes of differentially expressed miRNAs between the bacteria and bacteria+heat groups.

The target genes of all identified miRNAs were predicted using the miRanda algorithm, and there were total of 7696 pairing between protein-coding genes and miRNAs, which were equivalent to 5178 targets genes for 199 oyster miRNAs (Table S5). The target genes of 55 and 56 miRNAs were retrieved expressed differently in the control/bacteria and bacteria/bacteria+heat comparisons, respectively, and then assigned GO terms by Blast2GO software. The GO annotation of target genes were parsed and displayed through WEGO (Level-2) (Fig. 7). These GO annotation included 14 terms in Cellular Component ontology, 13 terms in Molecular Function ontology, and 23 terms in Biological Process ontology. Some terms (including antioxidant, cell killing, death, immune system process and response to stimulus) were correlated obviously with the physiological status of oyster exposure to bacteria challenge or heat stress. Furthermore, the term chemoattractant was observed in the comparison of the bacteria and bacteria+heat library, while not in the comparison of the control and bacteria library.

## Discussion

MiRNAs represent a class of small non-coding RNAs that repress gene expression on a post-transcriptional level by the degradation or translation inhibition of target gene mRNA. It is well-known that miRNAs can modulate several physiologic and pathologic processes, including host immune response and stress response [26], [27]. In the present study, three small RNA libraries were prepared from haemocytes of oysters after bacteria challenge and heat stress, and they were sequenced on the Illumina Hiseq2000 platform to identify the miRNAs in oyster. After the filtering of low-quality sequence, adaptor sequence and sequences shorter than 18 nt and longer than 30 nt, a total of 28,766,932 high-quality reads were obtained and the most abundant reads were 22 nt ones in all three libraries. The length distribution of reads in the present study was consistent with the reports from other metazoans [28]–[30], demonstrating that three small RNA libraries from oyster were technically reliable and suitable for the subsequent analysis. After the removal of other non-coding RNA, repeat sequence and mRNA degradation product, totally 199 miRNAs were identified in oyster haemocytes, among which 71 ones were known and 128 ones were novel. The number of oyster miRNAs identified in the present study was more than that of other molluscs including *H. rufescens* and *L. gigantean* [22], and the obvious difference might result from the distinct sequencing depth of small RNA library. The 128 novel miRNAs might be of mollusc-specific, because they were not mappable to any reported mature miRNAs in miRBase databases. Among all the miRNAs, cgi-miR-10a, cgi-miR-184b, cgi-miR-100, cgi-miR-1984 and cgi-miR-67a were top 5 most abundant ones in the control library. The expression of miR-184 and miR-10 was also observed to be higher in *Apostichopus japonicas* and *Plutella xylostella* [28], [31], and their higher expression in oyster haemocytes suggested that they might play a crucial role in the maintenance of haemocyte physiological function [13]. Both miR-100 and miR-67 were considered to be ancient and conserved ones because they had been observed in almost all of metazoans [13], [32], but miR-1984 might be mollusc-specific because they were just identified in the other two molluscs. Interestingly, 29 miRNAs from oyster were observed to have more than one coding region of the precursors, implying that they could be transcribed from multiply sites and have more promoters, which might endow them more activation approaches at the transcription level under the multiple conditions and more chances to be involved in the modulation of physiological function [33]. The discovery of oyster miRNAs demonstrated that there were conserved and mollusc-specific miRNAs in oyster, and those 199 miRNAs expressed in oyster haemocytes might be involved in the modulation of host immune response and stress response.

MiRNAs modulation has been considered as a critical regulatory principle in the vertebrate immunity and adaptation [8], [34]. In order to understand the potential role of miRNAs in oyster immune response, the expression profile of 199 miRNAs in haemocytes was analyzed after bacteria challenge. The overall expression level of oyster miRNAs in the bacteria library decreased in comparison with that in the control group. Among all the identified miRNAs, the expression level of 22 miRNAs increased significantly after bacteria challenge, while the expression of 33 miRNAs decreased significantly. The results indicated that the 55 miRNAs in oyster haemocytes were of immune-responsive, and they might function as essential regulators of immune response in oyster [35], [36]. The significantly different expression of these miRNAs could result from the activation of miRNA biogenesis or degradation pathway triggered by the immune response against bacteria challenge in oyster haemocytes [3], [37]. The target genes of the 55 immune-responsive miRNAs were annotated by immune-related GO terms including antioxidant, cell killing, death and immune system process. These immune-responsive miRNAs could be implicated in the immunomodulation, and they might regulate the expression of target genes at the post-transcriptional level to modulate redox reaction, phagocytosis and apoptosis of oyster. The immune regulation of these miRNAs had also been reported in other animals [38], [39]. For example, miR-184 was observed to implicate in the modulation of apoptosis in some metazoans [40]. The present results suggested collectively that immune challenge could induce the expression of immune-related miRNAs in haemocytes, and these induced miRNAs might modulate the immune response via the regulation of redox reaction, phagocytosis and apoptosis in oyster.

To further explore the immunomodulation mechanism of oyster miRNAs under stress exposure, the expression level of miRNAs in haemocytes was evaluated after the simultaneous treatment of bacteria challenge and heat stress. The overall expression abundance of oyster miRNAs in the bacteria+heat library was highest among the three libraries. Meanwhile, the expression of 65 miRNAs in the bacteria+heat library changed significantly (37 miRNAs increased and 28 miRNAs decreased) in comparison with that in the bacteria library, similar to the 8 heat-responsive miRNAs from *Brassica rapa* [41]. Among the 65 miRNAs, 42 miRNAs also changed significantly in the expression levels in the control/bacteria comparison, indicating that heat stress response might modulate the immune response of oyster through the expression regulation of immune-related miRNAs. Furthermore, other 23 miRNAs which did not change significantly in the expression level in the control/bacteria comparison might represent heat-specific ones in oyster haemocytes. The 65 differentially expressed miRNAs in the bacteria/bacteria+heat comparison could be annotated the chemoattractant term in the Molecular Function ontology, which was not observed in the control/bacteria comparison. These results suggested that the expression level of immune-related miRNAs in haemocytes could be modulated by heat stress response, and heat-related miRNAs might modulate the expression of chemoattractant-related genes under heat stress to improve the heat adaption.

There were 42 miRNAs whose expression level changed significantly in both control/bacteria and bacteria/bacteria+heat comparisons. The expression levels of these miRNAs were clustered to understand their potent regulatory function during bacteria challenge and heat stress. Among the 42 miRNAs, the expression level of 5 miRNAs (cgi-miR-2a, cgi-miR-307, cgi-miR-745b, cgi-miR-1984 and scaffold1144_2255) increased significantly, while 4 miRNAs (cgi-miR-10a, cgi-miR-10b, cgi-miR-182 and scaffold631_909) decreased significantly in both two comparisons. The similar expression mode of the 9 miRNAs demonstrated that bacteria challenge and heat stress had identical induction effect on their expression level. Heat stress could further improve the response of these miRNAs to bacteria challenge in oyster haemocytes and intensify their immunomodulation during the immune status. The target genes of the 9 miRNAs might be implicated in some physiological functions such as redox reaction and primary energy metabolism, which were essential for the immunity and stress response of mollusc [42]. Therefore, the expression alteration of these miRNAs after heat stress would not hamper the immune response of oysters against invasive pathogens, but further promote their adaption to the two environmental insults. However, the inconsistent expression of remaining 33 miRNAs in the two comparisons indicated that that heat stress could reverse the response of these miRNAs to bacteria challenge in oyster haemocytes and change accordingly their immunomodulation during the immune status. The reversed expression of the 33 miRNAs after heat stress might be detrimental to the immune response in oyster, because heat stress response could compete materials and energy with the immune response for host survival [43]–[45]. The results indicated that heat stress could modulate the immune response through the bilateral induction of immune-related miRNAs to keep maintain homeostasis and increase the adaption.

## Materials and Methods

### Ethics Statement

The oysters *C. gigas* (averaging 110 mm in shell height) used in the present study were marine cultured animals, and were collected from a local farm in Qingdao, Shandong Province, China, and maintained in the aerated seawater at 20°C for two weeks before processing. No specific permits are required for the described field studies, since the oysters in the local farm are provided for the local market-sellings. And the oyster *C. gigas* is not endangered or protected species. All the experiments were conducted according to the regulations of local and central government, and the study protocol was approved by the Experimental Animal Ethics Committee, Institute of Oceanology, Chinese Academy of Sciences, China.

### Bacteria Challenge and Heat Stress

One hundred and twenty oysters were employed for the bacteria challenge and heat stress experiment, and they were divided randomly into the control, bacteria and bacteria+heat groups. The oysters in the control and bacteria group received an injection of 100 µL phosphate buffered saline (PBS, 0.14 mol L^−1^ sodium chloride, 3 mmol L^−1^ potassium chloride, 8 mmol L^−1^ disodium hydrogenphosphatedodecahydrate, 1.5 mmol L^−1^ potassium phosphate monobasic, pH 7.4) and 100 µL suspension of live *Vibrio splendidus* strain (1×10^7^ CFU ml^−1^ in PBS), respectively, and maintained in the seawater at 20°C. The oysters in the bacteria+heat group were transferred to 28°C seawater after receiving an injection of 100 µL suspension of live *V. splendidus* strain (1×10^7^ CFU ml^−1^, in PBS). All individuals were sampled at 12 h post-injection, and the haemolymph collected from ten individuals were pooled into one sample in each group with three replicates (N = 3) for deep sequencing of small RNA libraries. Meanwhile, five individuals in each group were sampled randomly to collect the haemolymph, and there were five biological replicates (N = 5) for subsequent quantitative real-time PCR analysis of differentially expressed miRNAs. Haemocytes were harvested after the centrifugation at 800× g, 4°C for 10 min, and then resuspended and stored in liquid nitrogen immediately for RNA extraction.

### The Construction and Deep Sequencing of Small RNA Libraries

The low molecular weight RNA from the haemocytes of scallop in the control, bacteria and bacteria+heat groups was extracted using a mirVana™ miRNA isolation kit (Ambion, Austin, TX, USA) followed the manufacturer’s protocol. The total RNA in each haemocyte samples was quantified by Nanodrop 2000 (Thermo Scientific) at 260/280 nm (ratio >2.0) and its integrity was checked with Angilent 2100 Bioanalyzer (Agilent Technologies). For the library preparation of each group, the total RNA from 3 samples was mixed equivalently into a RNA sample (∼200 µg) for three technique repeats, and then it was size-fractionated on a 15% tris-borate-EDTA-Urea polyacrylamide gel. After the RNA fragments of 20–30 nucleotides were isolated, the Illumina proprietary adapters were ligated to their 5′ and 3′ terminals, following by reverse transcription. The three generated small cDNA libraries were amplified by PCR with primers complementary to the adaptor sequences. Subsequently, the libraries were deep sequenced by Illumina Hiseq2000 according to the manufacturer’s instructions (Genergy biotechnology company, ShangHai, China). The raw sequencing reads have been submitted to NCBI Short Read Archive under the accession number of SRR1066790–1066792.

### The Identification of Oyster miRNAs

Raw reads obtained from Illumina sequencing were processed by the Fastx-toolkit pipeline (http://hannonlab.cshl.edu/fastx_toolkit/index.html) to summarize data production, evaluate sequencing quality, remove low quality reads and adaptor sequences, and calculate the length distribution of small RNA reads [46], [47]. Small RNAs ranging from 18–30 nt in the small RNA library were collected for the removal of redundancy. The resulting clean reads mapped to the RepBase (www.girinst.org/repbase) and Rfam (http://rfam.sanger.ac.uk) were removed before further analysis. And the clean reads mapped to protein-coding mRNA sequences in oyster *C. gigas* were considered to be degradation products, and therefore, eliminated [31].

Oyster genomic sequences and annotation files are available and downloaded from the Comprehensive Library for Modern Biotechnology (CLiMB) repository (doi:10.5524/100030). After the filtered clean reads were mapped to mature miRNA in miRBase (version 19), the mappable reads were aligned to the oyster genome using bowtie software (http://bowtie-bio.sourceforge.net/index.shtml), and the aligned reads were further analyzed by miRDeep2 software (http://www.mdc-berlin.de/en/research/research_teams/systems_biology_of_gene_regulatory_elements/projects/miRDeep/) with the prediction of hairpin structure and precursor sequence to identify the known miRNAs in oyster. Meanwhile, the unmappable reads with registered mature miRNAs were also analyzed by miRDeep2 software to identify potential novel miRNAs in oyster. Multiple alignment of precursor sequences was performed with the ClustalW multiple alignment program (http://www.ebi.ac.uk/clustalw/).

### Expression Analysis of Oyster miRNAs

The copy numbers of known and novel miRNAs in three small RNA libraries were counted using a home-made Perl script. To determine the significant difference of miRNA copy numbers among the three small RNA libraries, the IDEG6 program (http://telethon.bio.unipd.it/bioinfo/IDEG6_form/) was utilized to perform a normalization calculation. Results of the Audice-Claverie test, Fisher exact test and Chi-squared 2×2 test with a Bonferroni correction for multiple comparisons, and a p-value <0.00001 indicated that differences in the miRNA copy numbers were statistically significant [48]. The expression levels of oyster miRNAs in each library were estimated using FPKM method, and a cluster dendrogram of oyster miRNAs with significant expression difference was constructed based on their expression levels using cluster 3.0 software and Treeview software.

### Quantitative Real-time PCR Analysis of Differentially Expressed miRNAs

Total RNA including miRNAs in oyster haemocytes were extracted and purified using miRNeasy Mini Kit (QIAGEN) according to its protocol. The reverse transcription of miRNAs was carried out based on QIAGEN miScript II RT Usage information using total RNA as template. The synthesis reaction was performed at 37°C for 1 h, terminated by heating at 95°C for 5 min. The cDNA mix was diluted with the addition of 200 µL RNase-free water for subsequent SYBR Green fluorescent quantitative real-time PCR.

The quantitative real-time PCR was carried out in a total volume of 25.0 µL, containing 12.5 µL of 2x QuantiTect SYBR Green PCR Master Mix (QIAGEN, miScript SYBR Green PCR Kit), 2.5 µL of diluted cDNA, 2.5 µL of each primers (10 mmol L^−1^), and 5.0 µL of RNase-free water. Eight miRNA fragments were amplified using specific forward primers (Table 3) and universal reverse primers, and the universal reverse primers and 5S primer (Table 3) were used to amplify 5S fragment as an internal control to verify the successful reverse transcription and calibrate the DNA template. The SYBR Green real-time PCR assay was carried out for each sample with three technical replicates in an ABI PRISM 7500 Sequence Detection System (Applied Biosystems). All data was given in terms of relative miRNA expression using the 2^−△△Ct^ method. The data was subjected to one-way analysis of variance (one-way ANOVA) followed by a multiple comparison. Differences were considered significant at *P*<0.05.

**Table 3:** Sequence of the primers used in the experiment.

Primer	Sequence (5′-3′)	Sequence information
P1	TAATACTGTCAGGTAAAGATGTC	Real-time cgi-miR-8 primer
P2	TGAGTATTACATCAGGTACTGA	Real-time cgi-miR-12 primer
P3	AACCCGTAGATCCGAACTTGTG	Real-time cgi-miR-100 primer
P4	TCCCTGAGACCATAACTTGTGA	Real-time cgi-miR-125 primer
P5	TGCCCTATCCGTCAGTCGCTGC	Real-time cgi-miR-1984 primer
P6	AGCTATAATGGTTGTCATTTGTA	Real-time scaffold631_909 primer
P7	CTTGGCACTGTCTGAGCGCAGGT	Real-time scaffold42648_5080 primer
P8	TTGAATTTATTGGTTGGGTGT	Real-time scaffold1599_5643 primer
P9	CAAGGATGACACGCAAAT	Real-time 5S primer

### Target Prediction and GO Analysis

The 3′UTR sequences of oyster protein-coding genes were retrieved based on the oyster genomic sequences and annotation information. The target genes of oyster miRNAs were predicted using the miRanda algorithm (score > = 160, free energy< = −25 kcal/mol, http://www.microrna.org/microrna/getDownloads.do) [49].

For GO analysis, all protein sequences of oyster were aligned by local blastp search to the non-redundant database of NCBI with *E*-value <1E-5, and then the alignment results were parsed by Blast2GO software (http://www.blast2go.com/b2ghome) for assigning GO terms. The GO terms of genes targeted by the differentially expressed miRNAs were calculated and exhibited through Web Gene Ontology Annotation Plot (WEGO, http://wego.genomics.org.cn/cgi-bin/wego/index.pl).

## Supporting Information

Table S1 The sequence and position information of known miRNAs in oyster.
(XLSX)

Table S2 The sequence and position information of novel miRNAs in oyster.
(XLSX)

Table S3 The reads numbers and expression levels of oyster miRNAs in control, bacteria and bacteria+heat libraries.
(XLSX)

Table S4 The information of differentially expressed miRNAs in the control/bacteria and bacteria/bacteria+heat comparisons.
(XLSX)

Table S5 All oyster miRNAs and their predicted target genes.
(XLSX)
